# OPPORTUNITIES AND CHALLENGES IN LARGE QUALITATIVE DATASETS IN GERONTOLOGICAL RESEARCH

**DOI:** 10.1093/geroni/igad104.0984

**Published:** 2023-12-21

**Authors:** Angela Perone, Pamela Saunders

## Abstract

Qualitative research focuses on in-depth data to understand people, processes, and phenomena. It often involves smaller datasets that do not intend to produce generalizable results but instead illuminate nuances, counterexamples, or underrepresented voices. Qualitative data can produce theory and allow for in-depth analyses. Recent methodological developments have created opportunities for large mixed methods projects to produce voluminous amounts of qualitative data that can and sometimes are shared as secondary datasets. The growing use of big data has also produced new possibilities for large qualitative datasets. Additionally, longitudinal and comparative qualitative research can produce significant data. This symposium presents four studies that examine opportunities and challenges in large qualitative datasets in gerontology. The first article uses multiple qualitative datasets to examine how long-term care staff balance resident rights of safety and autonomy. The second article uses multiple qualitative methods in a larger mixed methods Friendship Study to examine meaningful connections among people living with dementia in long-term care. Through a multi-site community university partnership, the third study extracts qualitative data from over 500 patient records to examine experiences of terminally ill hospice patients from residential homes for the dying. The fourth study examines nearly ten years of qualitative data from over 20 communities to examine successful aging from an Alaska Native perspective. Altogether, these four studies illuminate diverse strategies for identifying qualitative data, using data software, managing data, addressing ethical dilemmas, and employing research teams for coding and analyzing large qualitative datasets in gerontological research. This is a Qualitative Research Interest Group Sponsored Symposium.

